# Quality of anti-malarials collected in the private and informal sectors in Guyana and Suriname

**DOI:** 10.1186/1475-2875-11-203

**Published:** 2012-06-15

**Authors:** Lawrence Evans, Veerle Coignez, Adrian Barojas, Daniel Bempong, Sanford Bradby, Yanga Dijiba, Makeida James, Gustavo Bretas, Malti Adhin, Nicolas Ceron, Alison Hinds-Semple, Kennedy Chibwe, Patrick Lukulay, Victor Pribluda

**Affiliations:** 1 Promoting the Quality of Medicines Program, United States Pharmacopeia, Rockville, MD, 20852, USA; 2 Pan American Health Organization–Ecuador, Quito, Ecuador; 3 Department of Medical Chemistry, Anton de Kom (ADEK) University of Suriname, Paramaribo, Suriname; 4 Pan American Health Organization–Guyana, Georgetown, Guyana; 5 Food and Drug Department, Ministry of Health, Georgetown, Guyana

## Abstract

**Background:**

Despite a significant reduction in the number of malaria cases in Guyana and Suriname, this disease remains a major problem in the interior of both countries, especially in areas with gold mining and logging operations, where malaria is endemic. National malaria control programmes in these countries provide treatment to patients with medicines that are procured and distributed through regulated processes in the public sector. However, availability to medicines in licensed facilities (private sector) and unlicensed facilities (informal sector) is common, posing the risk of access to and use of non-recommended treatments and/or poor quality products.

**Methods:**

To assess the quality of circulating anti-malarial medicines, samples were purchased in the private and informal sectors of Guyana and Suriname in 2009. The sampling sites were selected based on epidemiological data and/or distance from health facilities. Samples were analysed for identity, content, dissolution or disintegration, impurities, and uniformity of dosage units or weight variation according to manufacturer, pharmacopeial, or other validated method.

**Results:**

Quality issues were observed in 45 of 77 (58%) anti-malarial medicines sampled in Guyana of which 30 failed visual & physical inspection and 18 failed quality control tests. The proportion of monotherapy and ACT medicines failing quality control tests was 43% (13/30) and 11% (5/47) respectively. A higher proportion of medicines sampled from the private sector 34% (11/32) failed quality control tests *versus* 16% (7/45) in the informal sector. In Suriname, 58 medicines were sampled, of which 50 (86%) were Artecom®, the fixed-dose combination of piperaquine-dihydroartemisinin-trimethoprim co-blistered with a primaquine phosphate tablet. All Artecom samples were found to lack a label claim for primaquine, thus failing visual and physical inspection.

**Conclusions:**

The findings of the studies in both countries point to significant problems with the quality of anti-malarial medicines available in private and informal sector facilities as well as the availability of therapy not compliant with national treatment guidelines. They also stress the need to strengthen regulatory control efforts on the availability of anti-malarial medicines in these sectors and in endemic areas.

## Background

Malaria transmission in the Amazon Basin of South America has fallen dramatically in the past 10 years. The malaria control programmes in Suriname and Guyana have made tremendous strides in reducing malaria transmission, especially in Suriname where the implementation of various strategies has lead to pre-elimination [1,2]. Suriname has had a 92% reduction in the transmission of *Plasmodium falciparum* malaria when comparing 2008 with 2000 and Guyana has experienced a reduction in reported malaria cases from 2005 to 2007 [3]. However, malaria continues to be a major public health problem in the interior of Guyana and Suriname, where gold mining and logging are the major occupations and access to public sector facilities is minimal due to a limited road system and rugged terrain. In these areas, febrile illnesses are usually assumed to be malaria and treatment is first sought in private sector facilities – licensed pharmacies, wholesalers and distributors and informal sector facilities – unlicensed shops and conveniences stores [3]. For gold-miners, who are paid by the day, accurate diagnosis and appropriate treatment for a suspected malarial illness are secondary to rapid relief of symptoms and an early return to work since absenteeism due to illness can severely impact their wages. The cost of these medicines is not a major consideration for most gold miners, and local shop owners usually carry a wide range of anti-malarials from a variety of sources, including more expensive artemisinin monotherapy and artemisinin-based combination therapy (ACT) [4].

Cases of counterfeit and substandard essential medicines have been reported in South America [5,6]. However, little is known about the quality of anti-malarials in the private and informal sectors because most malaria medicine-quality monitoring (MQM) activities in the region have focused on the public sector [7]. Therefore, these studies were performed in Guyana and Suriname, where private and informal sectors are prevalent, to assess the quality of anti-malarial medicines. The goal was to determine the quality of the collected anti-malarial medicines using pharmacopeial or other validated methods, thus providing evidence for targeted interventions. Similar studies of anti-malarials in the private and informal sectors in Southeast Asia and Africa have shown high frequencies of poor quality medicines [8-15].

The studies described in this report took place in the context of the Amazon Malaria Initiative (AMI), a seven-country regional programme established by the United States Agency for International Development (USAID), which was launched in 2001. The report also contains many of the attributes recommended in the Medicine Quality Assessment Reporting Guidelines [MEDQUARG] developed to improve the reporting of medicine quality data [16].

## Methods

The studies consisted of four phases: (1) identification of the sampling sites, in consultation with health professionals in the country; (2) training of personnel in sampling and data recording; (3) collection of medicine samples; and, (4) sample analysis (quality control testing) and review of results. The testing of the samples was carried out at the United States Pharmacopeia (USP) laboratory in Rockville, Maryland, and at a collaborating laboratory, the Centro Nacional de Control de Calidad, Instituto Nacional de Salud, Ministerio de Salud Pública, Peru. Both laboratories operate in compliance with internationally recognized standards and have ISO: IEC 17025:2005 accreditation.

### Selection of sampling sites

The sites for medicine sampling were selected in consultation with national malaria control programme authorities and regional Ministry of Health personnel. Selection was based on the level of mining or logging activity in the area, consultation with regional health personnel, malaria prevalence, population density, and/or distance from public health clinics located within a region.

**Figure 1:**
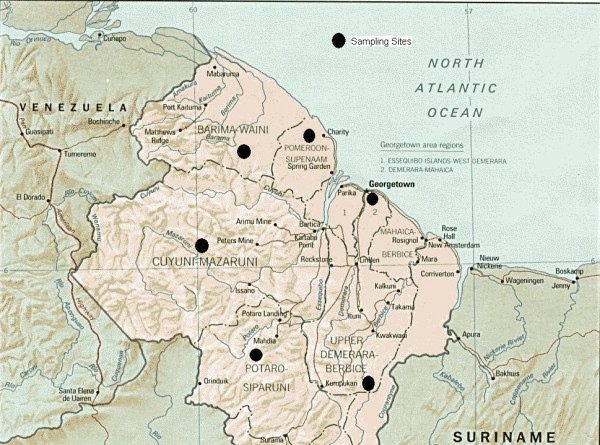
Map of sampling sites in Guyana.

In Guyana the study focused on six of its 10 regions: Barima-Waini (Region 1), Pomeroon-Supenaam (Region 2), Demerara-Mahaica (Region 4), Cuyuni-Mazaruni (Region 7), Potara-Siparuni (Region 8) and Upper Demerara-Berbice (Region 10) (Figure 1). Regions 1, 7 and 8 were selected because of their high malaria prevalence and the fact that they are home to numerous gold-mining operations, an activity that creates the environment for the breeding of the vector and exposes workers to infection. Regions 2, 4 and 10 were “source” regions, selected because miners, shopkeepers, and others often purchase anti-malarial medicines in the urban centres of these regions, before travelling to the more remote malarious areas.

**Figure 2:**
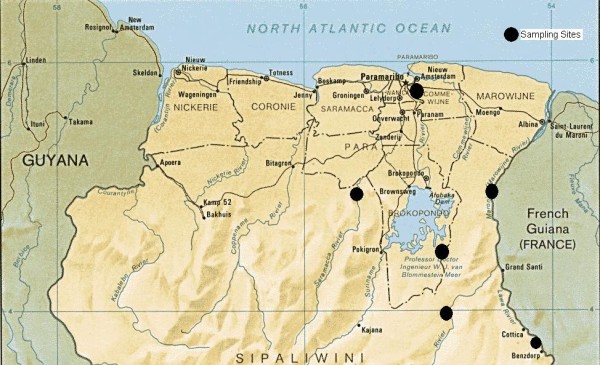
Map of sampling sites in Suriname.

In Suriname, the sampling scheme essentially followed the supply lines for the mining camps, which are based around the river system - the primary route of travel in these isolated regions (Figure 2). These areas include the Lawa River, Brokopondo Lake, Saramacca River, Marowijine River, and the area around the Tapanahony River in the eastern part of the country. Samples were also collected from the capital city of Paramaribo.

### Training and sampling

Local workers were trained utilizing standard sampling guidelines and implemented overt and mystery shopping techniques to collect samples [17]. Collectors were instructed to purchase the medicines in their original packaging; for medicines sold in bulk where that was not possible, they were asked to gather as much information as possible from the vendor regarding the name of the medicine, strength, expiry date, name of the manufacturer, and country of origin. All the relevant information was recorded on a standardized form as soon as possible after leaving the point of sale and before performing the next purchase.

The objective was to collect between 60 and 100 samples per country and a minimum of five units per sample (a unit is the individual tablet or capsule in a sample). All samples collected were handled to ensure sample integrity and avoid contamination. In Guyana, the data collectors were instructed to sample as many different anti-malarial medicines as possible at each site with priority given to artemisinin monotherapy or combinations. On the other hand in Suriname, most of the samples were of one particular ACT, Artecom, which was the most prevalent medicine identified.

### Quality control testing

All medicines underwent visual and physical inspection prior to quality control testing. A QC test failure was defined as a medicine failing any single QC test (i.e., identification, content, dissolution and etc.) for which it was evaluated. Overall failures were based on QC test and/or visual & physical inspection failures. A sample failing both a QC test and visual & physical inspection was considered as a single failure.

The quality of amodiaquine (AQ), primaquine (P), quinine (Q) and chloroquine (C) solid dosage forms was evaluated according to their individual monographs in the *USP-NF*[18]. Halofantrine and artesunate tablets were analysed following monographs in the *British* and *International Pharmacopoeia*s respectively [19,20]. The quality of mefloquine tablets was determined following the USP non-U.S. monograph [21]. ACT was evaluated by pharmacopeial, journal published, and manufacturers’ validated methods and pharmacopeial specifications for single medicines after they were determined to be suitable for use. The methods used in ACT analysis are given in Table 1. The dihydroartemisinin (DHA) content in Artecom and Artemos-40 was determined using a validated procedure provided by the Vietnamese National Institute of Drug Quality. The procedures to determine the content of sulphamethoxypyrazine (SMP) and pyrimethamine (PM) in Co-Arinate were obtained from the manufacturer.

**Table 1:** ACT analytical methods

**ACT**	**Active Pharmaceutical Ingredients in ACT**
Artecom	· dihydroartemisinin (DHA)
	· piperaquine (PPQ) [22]
	· trimethoprim (TMP) [18]
	· primaquine (P) [18]
Artemos-40	· dihydroartemisinin (DHA)
	· piperaquine (PPQ) [22]
	· trimethoprim (TMP) [18]
Co-Arinate	· artesunate (AS) [20]
	· sulphamethoxypyrazine (SMP)
	· pyrimethamine (PM)
Artemos-Plus	· artemether (A) [23]
	· lumefantrine (L) [24]

Due to limited sample availability, not all specified tests could be conducted on a sample therefore the type of tests performed depended on the number of units collected. In cases where the number of sample units was limited, identification (ID) and content/assay tests were given priority, followed by disintegration or dissolution testing. The Artecom and Artemos-40 samples were evaluated for disintegration since no suitable dissolution test was found. The Co-Arinate samples were evaluated for the dissolution of sulphamethoxypyrazine and pyrimethamine. Artemos-Plus samples were the only ACT evaluated for impurity content as these were the only products for which suitable impurity procedures were available. In addition, uniformity of dosage units (UDU) or weight variation (WV) was performed on all samples. There were six expired medicine samples (four were expired at time of sample collection and two expired before analysis): quality control analysis was limited to identification in this case, except for one sample, which was also evaluated for content. Due to the labour-intensive nature involved in the analysis of Artecom, a subset of those collected in Suriname was selected for evaluation. Expired medicines were excluded and not more than two samples with the same lot number but collected at different sites were analysed.

## Results

### Distribution, prevalence, source, origin and packaging

A total of 77 anti-malarial medicine samples collected between June and August 2009 from six regions of Guyana were analysed for quality. Of the 77 samples, 46 (60%) came from three “malaria endemic” regions (1, 7 and 8), and 31 (40%) from “source” regions (2, 4 and 10). The samples had been collected from 45 premises, 16 of which were private sector facilities and 29 were informal sector facilities. The majority of the samples (58%) were collected from the informal sector while 48% were obtained from facilities in the private sector.

More than half of the samples 47/77 (61%) were ACT, and 30/77 (39%) were monotherapy, that also included non-artemisinin derivatives, such as chloroquine and primaquine, the nationally recommended treatments for *Plasmodium vivax* in Suriname and Guyana. Dihydroartemisinin was the dominant artemisinin derivative found among ACT samples while the fixed dose combination of artesunate, sulphamethoxypyrazine and pyrimethamine was the second most widely encountered. Only one sample contained artemether. No samples of the fixed dose combination of lumefantrine – artemether, the nationally recommended treatment for *P. falciparum* were collected in either country. It is noteworthy that artesunate was also found among the monotherapy samples: two of the three artesunate samples came from a private pharmacy, the other an illegal vendor.

Based on the sampling results, the informal sector sells primarily ACT. Only 24% of the samples collected in this sector were monotherapy. This is in sharp contrast to the private sector where 59% (19/32) of the samples were monotherapy. Two ACT in particular, Artecom and Co-Arinate, were found to be very popular in the informal sector; though they were also present in a lesser extent in private sector pharmacies. Together Artecom (26 samples, 20 in the informal and 6 in the private sector) and Co-Arinate (19 samples, 14 in the informal and 5 in the private sector) represented 59% of all samples collected.

Most of the samples collected (63/77 or 82%) were obtained in their original individual container and included the package inserts. The other 14 samples (seven private sector and seven informal sector samples) were obtained from bulk containers, including unlabelled ones. Storage conditions in the informal sector were often unsatisfactory, with medicines kept under counters and in cupboards at very high ambient temperatures. In four cases, two in the private and two in the informal sectors, medicines were stored in unlabelled containers and no information could be obtained regarding the source or expiry date. Sampled medicines were labeled as manufactured in China (44%), Italy (24%), Cyprus (10%), Guyana (6%), India (5%), Pakistan (3%) and Switzerland (3%); the manufacturer was unknown for 5% of the medicines.

In Suriname, a total of 57 samples were collected from informal sector facilities in the Marowijne, Paramaribo and Sipaliwini districts with the majority collected in Sipaliwini. Of the 57 samples, 86% (49/57) were Artecom. These samples were from the same manufacturer as those sampled in Guyana; they comprised a total of eight different lots, three of which were the same as those found in Guyana.

### Visual & physical inspection and quality control test results

Approximately 58% (45/77) of the anti-malarial medicines sampled in Guyana were found to be of poor quality (Table 2), meaning the medicine failed one of the quality control tests and/or visual and physical inspection. The 45 failed samples represented three of the four presentations of ACT and five of the seven different monotherapy. The majority of the samples tested failed the test for content. Among monotherapy, nine of the 30 samples collected failed the test for dissolution while samples of quinine (Q) and mefloquine (M) tablets were found to not contain API. All of the artesunate tablet medicines were found to be of poor quality, two samples failed test for content, dissolution and impurities with the other sample failing visual & physical inspection. Similar quality issues were also found for two chloroquine samples. In both cases the samples were from the same manufacturer and batch, indicative of manufacturing issues.

**Table 2:** Visual & physical and QC Tests Results - Guyana

**Medicine**	**No. of Medicines Analyzed**	**No. of Visual & Physical Inspection Failures**	**QC Test Failed**						**No. of QC Test Failures**^**1**^	**Overall Failures (%)**^**2**^
			**Identification**	**Content**	**Dissolution**	**Disintegration**	**Impurity**	**UDU/WV**		
Artemos-Plus	1	0	0	0	-	-	1	0	1	1 (100)
Co-Arinate	19	0	0	0	0	0	-	0	0	0 (0)
Artemos-40	1	0	0	1	-	0	-	1	1	1 (100)
Artecom	26	26	0	3	-	0	-	1	3	26 (100)
Amodiaquine	5	0	0	0	0	-	0	0	0	0 (0)
Artesunate	3	1	0	2	2	-	2	1	2	3(100)
Chloroquine	4	1	0	2	2	-	-	2	2	3 (75)
Halofantrine	2	0	0	0	-	-	-	0	0	0 (0)
Mefloquine	7	0	1	0	4	-	0	0	5	5 (71)
Primaquine	4	1	0	2	-	-	-	0	2	3 (75)
Quinine	5	1	1	0	1	-	0	0	2	3 (60)
**Total**	77	30 (39%)	2	10	9	0	3	5	18 (23%)	45 (58)

^1^ Some medicines failed more than one QC test.

^2^Medicines failing visual & physical inspection or QC test. A medicine failing both was considered as a single failure.

A dash “-“ indicates the test was not performed.

Among the 18 medicines failing quality control tests, five (28%) were ACT, including three Artecom samples. While three of 26 Artecom samples failed the content test for one of the FDC constituents, DHA, a greater problem was found with the co-blistered primaquine tablet. The primaquine content ranged from 12–15 mg of the free base however, the package label did not provide information regarding the strength of the primaquine tablet resulting in a failure by visual and physical inspection.

The failure rates were similar in the malaria endemic Regions 1, 7 and 8 (24%) and the non-endemic “source” regions 2, 4 and 10 (23%). The highest proportion of failures, 30%, was found in Region 4 but, Regions 7 and 8. also stood out with 29% of samples failing quality control testing (Table 3). The test results also show a difference in medicine quality risk between buying from the private sector, where 34% (11/32) of samples failed, and buying from the informal sector, where there was a failure rate of 16% (7/45).

**Table 3:** Quality control test results by region, sector and anti-malarial class - Guyana

Region	Failures	Failures by Sector		Failures by Class	
		Private	Informal	ACT	Monotherapy
1	18% (4/22)	-*	4	0	4
7	29% (5/17)	3	2	4	1
8	29% (2/7)	1	1	1	1
**Total**^**1**^	**24% (11/46)**	**4**	**7**	**5**	**6**
2	15% (2/13)	2	0	0	2
4	30% (3/10)	3	-*	0	3
10	25% (2/8)	2	0	0	2
**Total**^**2**^	**23% (7/31)**	**7**	**0**	**0**	**7**
**Total**	**23% (18/77)**	**34% (11/32)**	**16% (7/45)**	**11% (5/47)**	**43% (13/30)**

*No samples were collected in this sector in the indicated region.

^1^Totals for malaria endemic regions 1,7 and 8.

^2^Totals for “source” regions 2, 4 and 10.

The quality control test results for Artecom samples from Suriname mirrored the test results from Guyana. All Artecom samples lacked primaquine dosage strength information on the packaging, a labeling violation by most MRAs. As in the case of the Artecom samples from Guyana, compliance to a label claim could not be assessed for the Suriname samples.

## Discussion

The itinerant behaviour of people inhabiting the hinterland regions (indigenous peoples, miners, and loggers) and the great distances between camps and officially sanctioned public health care facilities are conducive to self-medication and purchase from alternative sources. In these malaria endemic areas, there is a lack of health care professionals correctly dispensing medicines, even in private pharmacies. Anti-malarials are often dispensed by a non-pharmacist in the pharmacies, without requiring a prescription. As for informal sector vendors, the incentive to sell anti-malarials from under the counter is high. The commercial value of the current combination medicines containing artemisinin derivatives is significant and the demand is there.

Many of the anti-malarial medicines sampled from the private and informal sectors were not registered nor were they part of the national standard treatment guidelines. Medicines from both sectors, and across the varied regions, were of poor quality and several were found to be expired. Artemisinin monotherapy were also available, yet WHO guidelines do not recommend their use as treatment for uncomplicated *P. falciparum* malaria.

In the context of quality problems, of particular note is the case of the ACT, Artecom, which was not registered in either Guyana or Suriname. Based on vendors’ accounts and sampling results, it was the most popular anti-malarial in the informal sector in both countries. Interestingly, the same lot number was found for samples collected in both Guyana and Suriname, suggesting the possibility of a common supply source and/or illegal cross-border commerce of malaria medicines. Also, the packaging did not include dosage strength information for the content of primaquine, making it impossible to assess content and violates labeling requirements established in pharmacopeias and by most MRAs. In addition, the lack of primaquine content poses a safety concern to glucose-6-phosphate dehydrogenase (G6PD) deficient individuals receiving malaria treatment [25].

Samples of quinine and mefloquine tablets collected were found to not contain API, which may raise the issue of counterfeit medicines. However, according to legislation in Guyana, when a medicine contains no active ingredient it is considered as substandard unless there is other incriminating evidence that proves the drug is counterfeit. Additional incriminating evidence (i.e., suspicious packaging) was not found to make such a claim. In addition, the medicines were both sampled from bulk containers, which brought into play the potential for human error.

Non-compliance to GMP in the production of malaria medicines has been shown to be a problem among domestic and international manufacturers [26]. These results indicate quality problems were observed among locally manufactured and foreign products. Both artesunate (foreign product) and chloroquine (domestic product) failed tests for content, dissolution and uniformity of dosage units, quality attributes that are sensitive to variations in manufacturing. Although regulatory authorities are more capable of addressing problems with local manufactures, most malaria medicines are procured from abroad. Limited human and financial resources hinder their abilities to conduct inspections of international manufacturers.

The test results obtained were shared with regulatory authorities in both countries. It is hoped that the findings from this and other published studies will help provide a basis for targeted interventions. If it is not feasible for the public sector to expand services, it is recommended that health authorities increase inspections, training and monitoring of staff in private sector pharmacies to improve dispensing practices. Furthermore, if it would prove unfeasible to promptly eliminate sales of anti-malarials in the informal sector, the following approaches could be considered: 1) expand programmes, such as the active-case detection component of the “Looking for Gold Finding Malaria” programme in Suriname sponsored by the Global Fund to Fight Aids, Tuberculosis and Malaria; 2) include selected vending sites or stores in a parallel training, monitoring and inspection programme in order to improve knowledge and dispensing of anti-malarials (*cf.* the model successfully introduced in Tanzania) [15]. Public awareness campaigns about the importance of good quality medicines and the risk of purchasing from the informal sector can also have an impact [27,28]. The aim is not only to protect patients from harm, but also to protect currently effective treatments and prevent the development of drug resistance.

## Conclusions

The Guyana results illustrate the persistent problem of poor quality medicines and other aspects of irrational use of anti-malarial medicines in countries, especially if the malaria-endemic regions are remote or difficult to access. As illustrated by the Guyana test results in particular, there is a greater chance of buying non-registered and/or poor quality products in private establishments than in informal ones.

Findings from these studies are similar to those that investigated the quality of medicines in the private and informal sectors in Africa and Southeast Asia, where there was also high incidence of poor quality anti-malarials [28-30]. Coupled with the ease with which the artemisinin-based products can be acquired and the availability of clinically inappropriate artemisinin-based monotherapy, these are a major cause for worry. Together, they not only risk patient safety but also present serious implications for the development of drug-resistant strains of the *Plasmodium* parasite. A novel treatment is not foreseeable in the near future, thus placing malaria treatment at risk globally.

## Competing interests

The authors declare that they have no competing interests.

## Authors’ contributions

VP and AB conceived the studies and participated in the design and coordination. MA was involved in the co-ordination and selection of sampling sites in Suriname. NC and AH were involved in the co-ordination and selection of sampling sites in Guyana. MJ and GB co-ordinated sample collection in Guyana and Suriname respectively. DB, YD and SB were responsible for laboratory analysis of the medicines at USP. LE, VC and VP performed data analysis and drafted the manuscript with additional input from PL and KC. All authors read and approved the final manuscript.
